# Novel Cellular Mechanisms for Neuroprotection in Ischemic Preconditioning: A View from Inside Organelles

**DOI:** 10.3389/fneur.2015.00115

**Published:** 2015-05-26

**Authors:** Maria Josè Sisalli, Lucio Annunziato, Antonella Scorziello

**Affiliations:** ^1^Division of Pharmacology, Department of Neuroscience, Reproductive and Odontostomatological Science, School of Medicine, Federico II University of Naples, Naples, Italy; ^2^Fondazione IRCSS SDN, Naples, Italy

**Keywords:** ischemic preconditioning, sodium calcium exchanger, mitochondria, neurons, calcium

## Abstract

Ischemic preconditioning represents an important adaptation mechanism of CNS, which results in its increased tolerance to the lethal cerebral ischemia. The molecular mechanisms responsible for the induction and maintenance of ischemic tolerance in the brain are complex and not yet completely clarified. In the last 10 years, great attention has been devoted to unravel the intracellular pathways activated by preconditioning and responsible for the establishing of the tolerant phenotype. Indeed, recent papers have been published supporting the hypothesis that mitochondria might act as master regulators of preconditioning-triggered endogenous neuroprotection due to their ability to control cytosolic calcium homeostasis. More interestingly, the demonstration that functional alterations in the ability of mitochondria and endoplasmic reticulum (ER) managing calcium homeostasis during ischemia, opened a new line of research focused to the role played by mitochondria and ER cross-talk in the pathogenesis of cerebral ischemia in order to identify new molecular mechanisms involved in the ischemic tolerance. In line with these findings and considering that the expression of the three isoforms of the sodium calcium exchanger (NCX), NCX1, NCX2, and NCX3, mainly responsible for the regulation of Ca^2+^ homeostasis, was reduced during cerebral ischemia, it was investigated whether these proteins might play a role in neuroprotection induced by ischemic tolerance. In this review, evidence supporting the involvement of ER and mitochondria interaction within the preconditioning paradigm will be provided. In particular, the key role played by NCXs in the regulation of Ca^2+^-homeostasis at the different subcellular compartments will be discussed as new molecular mechanism proposed for the establishing of ischemic tolerant phenotype.

## Introduction

Cerebral ischemia is a multifactorial and complex disease (1, 2). Indeed, the intracellular events activated by the loss of perfusion of the brain and responsible for neuronal damage range from impairment of intracellular homeostasis to mitochondrial dysfunction and free radical production (3–5). The complexity of these events explains the great discrepancy between the frequency of cerebral ischemic accidents and the lack of effective treatments able to inhibit or slow neuronal demise following the ischemic insult. Hence, the urgent need to identify new potential targets for the development of innovative therapeutic strategies able to defend the ischemic brain.

On these premises, in the recent years, the attention of the researchers focused on ischemic tolerance a phenomenon, also known as ischemic preconditioning (IPC), which consists of a sub-lethal anoxic insult that makes the tissue in which it occurs more resistant to a subsequent and potentially lethal ischemia (6–11). The relevance of this phenomenon is to correlate to the study of the endogenous mechanisms activated in neurons to allow cell survival after a sub-lethal ischemic stimulus. By this way, it is possible to identify new molecular targets useful to develop alternative therapeutic strategies to treat the ischemic disease. The great interest in the cerebral IPC and in the tolerance evoked by itself also comes from the similarity of this phenomenon with those clinical situations occurring in the human brain. Indeed, it is well known that transient ischemic attacks (TIAs) do not cause structural damage but appear to protect brain against a subsequent “stroke” (12, 13).

Therefore, IPC or ischemic tolerance of the brain lie in a natural adaptive process that can be mimicked by a variety of sub-lethal insults, such as transient hypoxia, spreading depression, oxidative stress, hyperthermia, or heat shock, and that increases the tissue tolerance to a subsequent, potentially lethal ischemia. This adaptive cytoprotection is a fundamental property of living cells, which allows them to survive to the exposure to potentially recurrent stressors. This phenomenon was clearly identified in the heart by Murry et al. (14) as preconditioning, or subsequently as ischemic tolerance, and in 1990 it was described also in the brain by Kitagawa et al. (15). Since then, it immediately attracted the interest of clinical and basic neuroscientists for several reasons. First, this biological process became widely recognized as a pertinent and effective experimental instrument to understand how the brain protects itself against ischemia, thereby providing an innovative approach for the discovery of novel neuroprotective strategies. Second, retrospective case-control studies showed a clinical correlate of the phenomenon discovered experimentally.

## Molecular Mechanisms Involved in Ischemic Preconditioning

Among the molecular mechanisms underlining ischemic tolerance, the substantial changes in gene expression play a crucial role, suggesting that preconditioning is able to stimulate a genomic reprograming of cells which in turn is responsible for the cytoprotection and cellular survival described in those tissues interested by the ischemic event (16, 17). The effects in the cell genome, occurring after IPC, represent the signature of the complex interplay of multiple signaling pathways. It is interesting to underline that they are highly specialized pathways in different cell types of the brain and concur to the cellular and systemic response activated in the tissue to combat the noxious stimulus. Indeed, it has been reported that hundreds of genes undergo upregulation or downregulation in response to IPC stimuli (18–20), and that these changes in gene expression differ between the harmful ischemic insult and the sub-lethal IPC. In particular, preconditioning seems to attenuate the response to ischemia (19), and the tolerance established modifies the expression of genes involved in the suppression of metabolic pathways, in the control of immune responses, in the modulation of ion-channel activity, and in the changes of blood coagulation parameters (19).

As recently reported, three phases, temporally consecutive of the preconditioning phenomenon have been identified as induction, transduction, and tolerance.

### Induction and transduction mechanisms of tolerance

The induction of tolerance requires that the preconditioning stimulus has to be recognized by molecular sensors as a sign of a more severe phenomenon that will occur. This implicated the identification of numerous types of sensors, including neurotransmitters, neuromodulators, cytokines, and toll-like receptors (21) as well as ion channels and redox-sensitive enzymes (22–24) which in turn will activate subsequent transduction pathways responsible for initiating the adaptive response. The activation of the transduction phase also implies the involvement of different transducers depending, at least in part, on the nature of the preconditioning stimulus. They include members of mitogen-activated protein kinases (MAPKs) and their phosphorylated Ras, Raf, MEK, and ERK subfamilies (7, 22, 24, 25); mitochondrial ATP-sensitive K+ (KATP) channels (26, 27) Akt (28–31); and the protein kinase C-ε isoform (32). The possibility that the nitric oxide (NO)-based adaptive response to hypoxia in *Drosophila* (33) is evolutionarily conserved suggests that this multifunctional modulator might be a logical choice as an autocrine and paracrine mediator of preconditioning stress. Indeed, pharmacological and genetic evidence supporting the involvement of NO in the transduction process is continuing to mount. As matter of fact, it has been reported that NO can exert a neuroprotective role during preconditioning (7, 34), and we have recently proposed that NO-induced neuroprotection is associated to preservation of mitochondrial function (24). On the other hand, preconditioning positively affects the integrity of mitochondrial oxidative phosphorylation after cerebral ischemia, an effect restricted to the delayed window of preconditioning (35). Moreover, preconditioning prevents mitochondrial swelling, preserves membrane integrity fluidity, and protects mitochondrial energy metabolism during cerebral ischemia avoiding ATP consumption (36).

Given the redox sensitivity of many of those above-mentioned kinases and transcription factors, reactive oxygen species might also be contemplated within transducers (37–39). Furthermore, adenosine, another prototypical paracrine mediator and “retaliatory metabolite,” whose production is linked to ATP degradation, might be considered a transducer responsible for tolerance induction, in some *in vitro* and *in vivo* models (26, 27, 40, 41).

It is worth of note that although in general, mitochondria have been considered important mediators of endogenous neuroprotection, the mechanisms by which they might “integrate” cytoprotective signaling of preconditioning remain to be fully elucidated.

### Role of mitochondria in ischemic tolerance

It is well known that mitochondria are also able to sense and shape cytosolic Ca^2+^ signals by taking up and subsequently releasing Ca^2+^ ions during physiological and pathological Ca^2+^ elevations (42). Mitochondrial function is strictly dependent on maintaining of mitochondrial calcium homeostasis. In fact, thanks to the activity of Ca^2+^-sensitive mitochondrial dehydrogenases these organelles can regulate oxidative phosphorylation and ATP synthesis during conditions of high cellular demand (43). Therefore, to explore the relationship between mitochondrial oxidative capacity and calcium buffering activity during IPC might represent an attractive strategy mediating neuroprotection in ischemic conditions. In particular, considering that the influence of Ca^2+^ in the regulation of mitochondrial function is highly dependent on the spatiotemporal distribution of [Ca^2+^]_i_ (44, 45), the assumption that during IPC an interaction between mitochondria and ER might affect mitochondrial metabolic properties, and in turn neuronal survival has been also investigated (31). This hypothesis is supported by the concept that microdomains of high [Ca^2+^]_i_ have been identified near Ca^2+^ channels on the plasma membrane and ER (46).

## Cellular Ionic Homeostasis and Energy Metabolism during Ischemic Preconditioning

It has been widely described that IPC activates intracellular biological responses prior to a potential lethal insult (17, 47–49), and that these events make the tissue in which they occur more resistant to the subsequent severe ischemia (17, 47, 50–52). In this regard, it is possible to speculate that an increase of energy metabolism or a latency in anoxic depolarization following the onset of ischemic insult might represent one of the mechanisms by which tissues strengthen their tolerance when exposed to a sub-lethal insult. Indeed, it has been reported that a reduction in energy demand and in the activity of ion channels represents determinant factors for ischemic tolerance establishment (19, 53, 54). To this aim, *in vitro* experiments performed in cortical neurons demonstrated that the exposure to brief non-injurious oxygen and glucose deprivation (OGD) causes an impairment in voltage-gated potassium channels (55). Similarly, the impairment of Na^+^/K^+^-ATPase activity occurring in rat hippocampal and cortical neurons exposed to global forebrain ischemia is counteracted by IPC (56). Interestingly, in the last years, it has been shown that some integral plasma membrane proteins, involved in the control of Ca^2+^ and Na^+^ ion influx or efflux, the sodium calcium exchangers (NCXs) might function as crucial players in the pathogenesis of brain ischemic damage (57). Therefore, these proteins, by regulating Na^+^ and Ca^2+^ homeostasis, may represent more suitable molecular targets for therapeutic intervention in ischemic stroke. As matter of fact, *in vivo* experiments performed in gerbils demonstrated that during IPC, an increase in Ca^2+-^ATPase activity and an enhancement in mitochondrial calcium sequestration occur in CA1 hippocampal neurons (47). In line with this findings, intracellular calcium measurements in hippocampal neurons of preconditioned gerbils showed that the increase in [Ca^2+^]_i_ occurring after anoxic and aglycemic episodes was markedly reduced in the ischemic tolerant animals (58). However, the molecular mechanisms responsible for this effect are still matter of debate and not completely clarified. As recently proposed by Kato et al. (59), a possible explanation could be related to the increased expression of the Ca^2+-^ATPase isoform 1 [plasma membrane calcium ATPase 1 (PMCA-1)]. Moreover, considering that mitochondria play a crucial role in the regulation of intracellular Ca^2+^ homeostasis due to their high capacity to sequestrate Ca^2+^ both in physiological and in pathological conditions, they have been included among the possible intracellular mechanisms triggered by preconditioning stimuli. Indeed, although it is widely reported that the excessive amount of Ca^2+^, as it occurs during ischemia, causes an impairment of mitochondrial function with consequent massive uncoupling of oxidative phosphorylation, mitochondrial depolarization and mitochondrial permeability transitional pore (MPTP) opening, reversal of the action of ATP synthase and cell death, and studies performed both in the heart and in the brain demonstrated that the inhibition of MPTP opening and its signaling cascade represent crucial events responsible for cytoprotection observed in IPC (53, 60). What are the molecular mechanisms underlining these effects is still object of investigation. Among them, nitrite and protein kinases have been proposed as possible MPTP regulators (61–64) Table 1.

**Table 1:** **Mitochondrial effectors of preconditioning-induced neuroprotection**.

Type of study	Species	Stimulus	Mechanism	Reference
*In vivo*	Rat	Ischemic	Energy metabolism and mitochondrial function	(35)
				(65)
*In vitro*	Hippocampal neurons	Ischemia	Mitochondrial ATP-dependent potassium channel	(66)
*In vivo*	Rat	
*In vivo*	Mouse	Hypoxia	GLUT-1	(18, 63)
	Rat			(67)
*In vivo*	Rat	Normobaric Hypoxia	Phosphofructokinase and LDH	(67)
*In vivo*	Rat	Ischemia	Calcium/calmodulin-dependent protein kinase II	(68, 69)
*In vivo*	Gerbil	Ischemia	Akt/protein kinase B	(28)
*In vivo*	Rat	Ischemia	mitogen-activated protein	(70)
*In vitro*	Cortical neurons	Hypoxia	NCX3	(31)
*In vitro*	Cortical neurons	Hypoxia	MnSOD	(24)
*In vivo*	Rat	Ischemia		(23)

More interestingly, the hypothesis that a modulation of the expression and activity of the NCXs might have a role in the regulation of calcium and sodium homeostasis during ischemic tolerance has been recently suggested (31, 50, 71). This might be relating to the ability of Na^+^/Ca^2+^ exchanger to work in concert with selective ion channels and ATP-dependent pumps, in maintaining the physiological cytosolic concentrations of these two ions (72).

### NCXs functional properties in ischemic brain

In the brain, unlike other tissues, NCX is present in three different gene products, named NCX1, NCX2, and NCX3, with a distinct distribution pattern in different brain regions (73). Under physiological conditions, NCX works primary extruding Ca^2+^ in response to a depolarization or to an increase in intracellular Ca^2+^ concentrations coupled to receptor stimulation (74). However, during hypoxic conditions, when the dysfunction of the two plasma membrane ATP-dependent pumps Na^+^/K^+^ ATPase and Ca^2+^ ATPase occurs, NCX assumes a relevant role in controlling the intracellular homeostasis of these two cations, since it is able to operate promoting Na^+^ ions extrusion and Ca^2+^ influx (74, 75). Although this reverse mode of operation in the early phase of anoxia does undoubtedly elicit an increase in [Ca^2+^]_i_, its effect could be beneficial for neurons because it contributes to decrease [Na^+^]_i_ overload, a phenomenon which would otherwise lead to cell swelling and thus to sudden necrotic neuronal death. Conversely, in the later phase of neuronal anoxia, when [Ca^2+^]_i_ overload takes place, NCX working in the forward mode of operation can contribute to the lowering of Ca^2+^ concentrations, and thus it can protect neurons from intracellular Ca^2+^overload, neurotoxicity, and subsequent cell death (74). As we recently demonstrated, NCX1, NCX2, and NCX3 may exert different roles during *in vitro* and *in vivo* anoxic conditions leading to a new paradigm in the pathogenesis of ischemic damage. Indeed, we provided evidences that in cells singly and stably transfected with NCX3, this isoform contributes more significantly to the maintenance of [Ca^2+^]_i_ homeostasis during experimental conditions mimicking ischemia, thereby preventing mitochondrial ΔΨ collapse and cell death (76). This is due to a different sensitivity of the three NCX isoforms to the changes in ATP content occurring during anoxia. Indeed, NCX3 results more resistant to ATP changes compared to NCX1 and NCX2 (76). In addition, in *in vivo* experiments, the selective knocking down of NCX1 and NCX3, but not of NCX2, by antisense oligodeoxynucleotide strategy (57) or the disruption of the *ncx3* gene, renders the brain more susceptible to the ischemic insult (77). Moreover, the induction of permanent middle cerebral artery occlusion in rats correlates with NCX1 mRNA upregulation in the peri-infarct area thus suggesting the possibility that this isoform could be a new druggable target for the treatment of cerebral ischemia. In line with this hypothesis, we recently demonstrated that NCX1 transcript and protein were upregulated by ischemic cerebral preconditioning. This effect was mediated by the transcriptional factor HIF1α and was accompanied by a relevant neuroprotection (71).

### Role of NCX1 and NCX3 in the ischemic preconditioning

**Figure 1:**
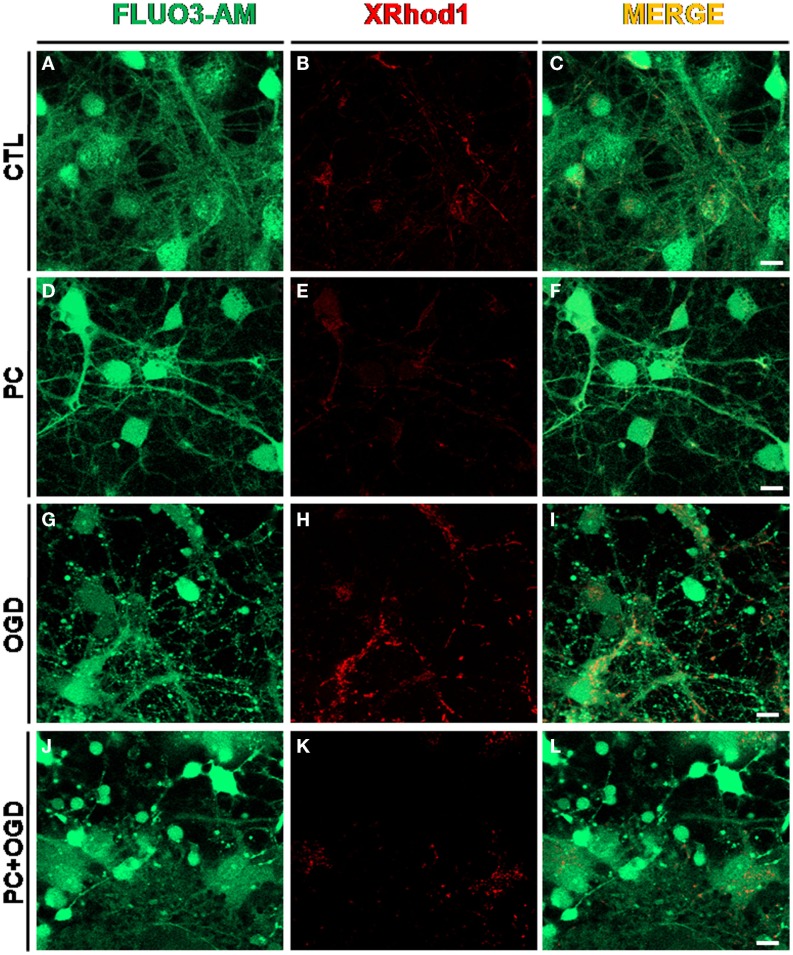
**Effect of ischemic preconditioning on cytosolic and mitochondrial calcium content in cortical neurons exposed to OGD**. Confocal images showing in red mitochondrial Ca^2+^ content measured by XRhod-1 probe (200 nM, 15 min at RT) and in green cytosolic calcium concentration measured by Fluo 3 AM probe (5 μM, 30 min at RT). **(A–C)**: cortical neurons under control conditions (CTL); **(D–F)**: cortical neurons exposed to 30 min of oxygen and glucose deprivation (OGD), preconditioning stimulus (PC); **(G–I)**: cortical neurons exposed to 3 h OGD; **(J-L)**: preconditioned neurons exposed to OGD (PC + OGD). Scale bars: 1 μm.

Recent findings demonstrated that the two isoforms of the Na^+^/Ca^2+^ exchanger, NCX1 and NCX3, which are involved in several pathophysiological aspects of cerebral ischemia, can be included among the members of the growing family of the mediators involved in the ischemic brain tolerance due to their ability to regulate neuronal calcium homeostasis (Figure 1). In particular, we provide evidence that neuroprotection observed in preconditioned neurons exposed to OGD/reoxygenation is correlated to the increase in NCX1 and NCX3 protein expression. Indeed, the treatment with siRNA against NCX1 and NCX3 prevents this effect. These data are in accordance with results recently observed *in vivo* in an animal model of IPC (50). Consistently with these results, the upregulation of NCX1 and NCX3 protein expression in neurons exposed to IPC was dependent on PI3K/Akt activation, since the treatment with LY294002 was able to abolish this increase. Interestingly, we demonstrated that NO plays a key role in the triggering PI3K/Akt pathway as the increase in the phosphorylated form of Akt observed within 30 min after IPC was completely abolished by the treatment with L-NAME. More importantly, the treatment with L-NAME was able to inhibit NCX3 but did not affect NCX1 protein expression (31).

## Calcium Homeostasis in ER and Mitochondria during Ischemic Preconditioning

**Figure 2:**
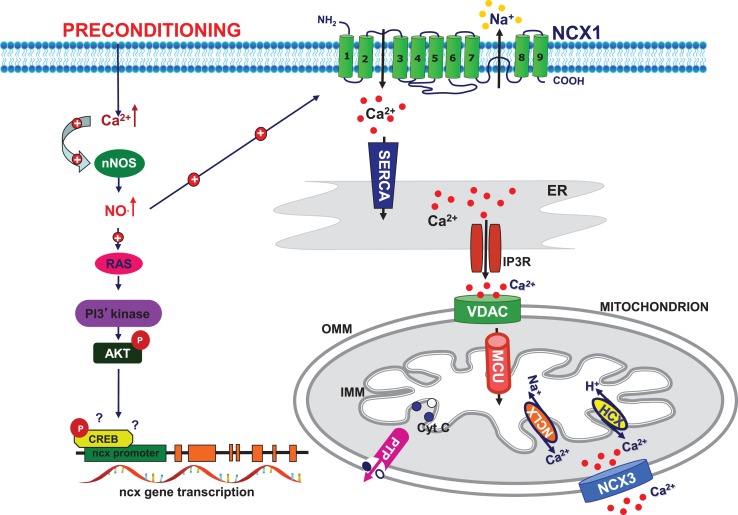
**Schematic model of preconditioning-induced neuroprotection**. Preconditioning stimulus activates neuronal nitric oxide synthase (nNOS) to produce NO which in turn stimulates plasma membrane NCX1 activity in the reverse mode of operation and promotes endoplasmic reticulum (ER) refilling in the early phase of PC. NO activate PI3K/Akt pathway, which increases the expression and activity of plasma membrane and mitochondrial NCX3, thus promoting mitochondrial calcium efflux in the late phase of preconditioning. These mechanisms working in concert promote neuronal survival. *PI3K:* phosphatidylinositol-3-kinases; *CREB:* cAMP response element-binding protein; *SERCA:* sarco/ER Ca^2+^-ATPase; *IP3R:* inositol trisphosphate receptor; *MCU:* mitochondrial calcium uniporter; *VDAC:* voltage-dependent anionic channel; *NCLX:* Na^+^/Ca^2+^ exchanger Li-dependent; HCX: Ca^2+^/H^+^ exchanger; *PTP:* permeability transition pore; *OMM*: outer mitochondrial membrane; *IMM:* inner mitochondrial membrane.

It has recently demonstrated that in neurons NCX3, apart its localization on the plasma membrane, is also expressed on the outer mitochondrial membrane where it contributes to the extrusion of calcium from mitochondria (78). It is well known that mitochondria, in addition to the generation of cellular energy, play an important role in regulating cellular calcium homeostasis (79–82) in concert with the sarco-endoplasmic reticulum Ca^2+^-ATPase (SERCA), the plasma membrane Ca^2+^-ATPase, and Na^+^/Ca^2+^ exchanger (83). On the other hand, the maintenance of mitochondrial calcium homeostasis is an important requirement ensuring mitochondrial function. In fact, Ca^2+^-sensitive dehydrogenases can regulate oxidative phosphorylation and ATP synthesis during times of high cellular demand (43). Therefore, it is possible to hypothesize that the increased expression of NCX3, we observed within 48 h from the sub-lethal insult, might exert neuroprotective effects regulating calcium handling and improving mitochondrial oxidative capacity. This finding is in line with the results previously obtained in an *in vitro* model of IPC and demonstrating that the increase in nNOS expression and NO^•^ production through the activation of Ras/ERK1/2 pathway stimulated mitochondrial MnSOD. These effects associate with a reduction in free radical production and cytocrome *c* release from mitochondria to cytosol, and in turn with an improvement of neuronal survival (24). The demonstration that NCX3 might represent a target of IPC-induced neuroprotection (31) adds new insight into the molecular mechanisms involved in the ischemic brain preconditioning. Indeed, the over-expression of NCX3 might help mitochondria to preserve their energetic capacity making them less vulnerable to the subsequent lethal insult represented by OGD/reoxygenation. This hypothesis was strongly supported by the results obtained measuring the activity of NCX during IPC. In fact, we demonstrated that IPC induced an increase of NCX activity in the reverse mode of operation that was still observed in preconditioned neurons exposed to OGD/Reoxygenation. This is an early event since it occurred within 30 min after IPC stimulus was due to the contribution of NCX1 and NCX3 isoforms and was promoted by NO because it was abolished by the treatment with L-NAME. Intriguingly, this effect was associated with an increase in ER calcium content. Consistently with data previously published in our Laboratory (84), we speculated that NCX1, working in the reverse mode of operation, plays a key role in the regulation of ER calcium refilling in the early phase of IPC (Figure 2). Indeed, the treatment with siNCX1, but not siNCX3, was able to prevent this phenomenon. The novel aspect of the study is the demonstration that NO promoted NCX1-induced ER refilling that was hampered by L-NAME pretreatment. This finding is in accordance with data recently published by Secondo et al. and demonstrating that NO was able to stimulate NCX1 to work in the reverse mode of operation, whereas NO did not affect NCX3 activity (85). The possibility that NCX1 activation in the early phase of IPC could affect mitochondrial calcium content promoting mitochondrial calcium uptake could not be excluded. However, in the late phase of IPC mitochondrial calcium handling is mainly regulated by NCX3 that is able to promote mitochondrial calcium extrusion (Figure 2). These data are supported by the finding that NCX3 expression increased 48 h after the IPC insult (31). We have previously demonstrated that NCX3 is distributed also at mitochondrial level; therefore, it is possible to speculate that during IPC, the increased expression of NCX3 on mitochondria might contribute to the efflux of calcium from the organelle thus protecting neurons by the subsequent mitochondrial calcium overload induced by lethal OGD/reoxygenation exposure. The finding that the treatment with CGP and siNCX3 counteracted the effect of IPC on mitochondrial calcium content, leading to the lack of IPC-neuroprotection, further supports this hypothesis (31). Collectively, we can conclude that a functional interplay between NCX1 and NCX3 occurs during IPC. This phenomenon is tightly dependent on NO and Akt activation, and by contributing to the modulation of intracellular ionic homeostasis, could represent one of the mechanisms responsible for neuroprotection induced by IPC (Figure 2).

## Conclusion

The experimental data described in this review support the hypothesis that mitochondria play a pivotal role in neuroprotective events provided by preconditioning. Indeed, both the mitochondrial antioxidant enzyme MnSOD and the proteins involved in the regulation of mitochondrial calcium homeostasis, such as mNCX3, represent new targets and mediators involved in preconditioning-induced neuroprotective responses. This is in line with the results reported in the literature that “mitochondrial preconditioning” has many neuroprotective effects, both during and following neurotoxic insults, including an improvement of neuronal viability, the attenuation of the intracellular Ca^2+^ influx, the suppression of ROS generation, the inhibition of apoptosis, and the maintenance of ATP levels (86). Therefore, understanding the precise mitochondrial mechanisms involved in preconditioning will provide important information necessary to develop new and more effective therapeutic strategies for neurodegeneration occurring in brain ischemia. In this regards, the identification of NCX3 as new player in the regulation of mitochondrial calcium efflux and the demonstration that an increase in its expression occurs during preconditioning, promoting mitochondrial calcium homeostasis, might help to draw new therapeutic strategy potentially able to delay neuronal loss occurring in ischemia. More interestingly, the demonstration that an increase in NCX1 and NCX3 activity is responsible for Ca^2+^ cycling from ER and mitochondria, and proof that it activates intracellular events leading to neuroprotection observed in IPC support this hypothesis (31). In this scenario, the identification of a compound able to selectively stimulate the activity of NCX1 and to prevent neuronal degeneration in *in vitro* and *in vivo* models of ischemia has been recently synthesized (77). Moreover, compounds able to stimulate NCX3 activity are in progress of development in our Laboratory.

Therefore, based on this early observation, these studies have potentially revealed new molecular targets in cerebral ischemia and neurodegenerative diseases pathogenesis, which ultimately may open up alternative avenues for future therapeutic intervention.

## Conflict of Interest Statement

The authors declare that the research was conducted in the absence of any commercial or financial relationships that could be construed as a potential conflict of interest.
